# Light-Induced Smooth Endoplasmic Reticulum Rearrangement in a Unique Interlaced Compartmental Pattern in *Macaca mulatta* RPE

**DOI:** 10.1167/iovs.62.15.32

**Published:** 2021-12-30

**Authors:** Annalisa Altera, Virginia Barone, Ivanela Kondova, Jan A. M. Langermans, Mariangela Gentile, Carmen Pin, Claudio Nicoletti, Eugenio Bertelli

**Affiliations:** 1 Department of Life Sciences, University of Siena, Siena, Italy; 2 Department of Molecular and Developmental Medicine, University of Siena, Siena, Italy; 3 Division of Pathology and Microbiology, Animal Science Department, Biomedical Primate Research Centre, Rijswijk, the Netherlands; 4 Animal Science Department, Biomedical Primate Research Centre, Rijswijk, the Netherlands; 5 Department Population Health Sciences, Division Animals in Science and Society, Faculty of Veterinary Medicine, Utrecht University, Utrecht, the Netherlands; 6 Clinical Pharmacology and Safety Sciences, BioPharmaceuticals R&D, AstraZeneca, Cambridge, United Kingdom; 7 Department of Experimental and Clinical Medicine, University of Florence, Florence, Italy

**Keywords:** endoplasmic reticulum, retinal pigment epithelium, Rhesus monkey, light damage, oxidative stress

## Abstract

**Purpose:**

To investigate light-induced modifications of the smooth endoplasmic reticulum of the RPE in primates.

**Methods:**

Eyes of three terminally anesthetized Rhesus monkeys were exposed to 5000 lux for 10 minutes or kept in the dark. Transmission electron microscopy and electron tomography were conducted on small fragments of retina sampled from different regions of the retina.

**Results:**

RPE cells smooth endoplasmic reticulum shows a previously unknown arrangement characterized by an interlaced compartmental pattern (ICP). Electron tomograms and 3D-modelling demonstrated that the smooth endoplasmic reticulum with an ICP (ICPSER) consisted of four parallel, independent and interwoven networks of tubules arranged as interconnected coiled coils. Its architecture realized a compact labyrinthine structure of tightly packed tubules stabilized by intertubular filamentous tethers. On average, the ICPSER is present in about 14.6% of RPE cells. Although ICPSER was preferentially found in cells located in the peripheral and in the para/perifoveal retina, ICPSER cells significantly increased in number upon light exposure in the para/perifovea and in the fovea.

**Conclusions:**

An ICPSER is apparently a unique feature to primate RPE. Its rapid appearance in the area centralis of the retina upon light exposure suggests a function related to the foveate structure of primate retina or to the diurnal habits of animals that may require additional protection from photo-oxidation or enhanced requests of visual pigments regeneration.

Eyes are specialized sensory receptors that exploit light to explore the surrounding environment; however, light can be also detrimental to eyes. Indeed, excessive and/or prolonged light exposure induces retinal damage. Intense visible light in itself is a cause of retinal injury,^1^^,^^2^ and light exposure in the spectral blue range has been linked to AMD.^3^ Even the progression of some forms of RP is influenced by light exposure.^4^ Indeed, there is growing consensus that a common denominator in the pathogenic pathway of all these disorders is an increased oxidative stress as a major inducer of RPE cell damage.^2^^,^^3^^,^^5^^–^^7^ Excessive light exposure affects the morphology and function of several subcellular organelles, including mitochondria, peroxisomes, and the endocytic/lysosomal and autophagic pathways.^8^^,^^9^ For instance, light exposure evokes the formation of ring-shaped mitochondria.^10^^,^^11^ Peroxisomes, in contrast, elongate and proliferate when exposed to UV light,^12^ and light increases the number of autophagic events in RPE cells.^13^^–^^15^

The smooth endoplasmic reticulum (SER) is an organelle that serves several functions, including lipid synthesis, detoxification, calcium storage, and carbohydrate metabolism.^16^ It occupies a large part of the RPE cell cytoplasm where it is shaped as an irregular network of branched tubules.^17^^,^^18^ Less frequently, it can be found arranged to form myeloid bodies.^18^^–^^20^ To date, however, very little is known on the effects of light on the SER structure in the RPE because investigations have been restricted to amphibians.^18^^,^^21^

Thus, to further study light-induced SER modifications, we carried out an investigation on the SER in the RPE of the nonhuman primate *Macaca mulatta*. Surprisingly, we found that *Macaca mulatta* RPE cells are provided with a domain of the SER apparently unique to these cells, showing an interlaced compartmental pattern (ICP) consisting of four interwoven tightly packed distinct tubular networks. More importantly, SER with an ICP (ICPSER) seems to be a light-dependent SER domain as it rapidly builds up upon light exposure in the area centralis of the retina.

## Methods

### Animals and Tissues

Animals were obtained from the in-house NHP naturalistic breeding groups of the Biomedical Primate Research Centre and were >F2 generation. Animals were housed socially in dedicated animal facilities containing bedding that allowed foraging and that were equipped with nonfood enrichment items, such as resting places at different levels, swings, Kong toys, and mirrors. Food enrichment items, such as food puzzles and frozen fruits, were provided daily and drinking water was available ad libitum via automatic water dispensers. Animals were fed monkey chow (Ssniff, Soest, Germany) daily in the morning, complemented with vegetables, fruits, or grain mixtures in the afternoon. The animals used in this study were not euthanized to obtain their eyes, but for welfare and ethical reasons. The judgement for euthanasia and the possibility to perform this specific study was made by the institute's veterinarians, the colony manager, and the Animal Welfare Body. All procedures in this study were in accordance with the Dutch law on animals, the ARVO Statement for the Use of Animals in Ophthalmic and Vision Research, and the European legislation concerning the care and use of laboratory animals. The Biomedical Primate Research Centre has been accredited by the Association for Assessment and Accreditation of Laboratory Animal Care since 2012.

Four eyes were sampled from two adult 7- and 8-year-old male Indian-origin Rhesus monkeys (*Macaca mulatta*) R10019 and R10026. The animals were deeply anesthetized with a mixture of ketamine (15 mg/kg) and medetomidine (20 µg/kg) in their home cage and subsequently transferred to the surgery. Here, the animals received an additional intramuscular injection of buprenorphine (20 µg/kg). During the first 30 minutes of anesthesia, eyes of both anesthetized animals were kept closed and covered with a dark bandage. Subsequently, the eyes of animal R10026 were opened and exposed to an illumination of 5000 lux for 10 minutes as previously reported.^22^^–^^24^ After a recovery time of 30 minutes with the open eyes, the thoracic cavity was opened and a cannula was inserted through the left ventricle into the aorta. The descending aorta was clamped just above the diaphragm. Using a syringe pump (Type S2; Medima, Clarence, NY), animals were perfused through the aorta with 25.000 IU of heparin in cold saline. Using a syringe pump (type S2; Medima), animals were perfused through the aorta with 25,000 IU of heparin in cold saline until the out-coming perfusate was clear. Then, the syringe pump was switched to ice-cold 2% paraformaldehyde and 2% glutaraldehyde in 0.1 M phosphate buffer, pH 7.4, and perfusion with about 1.5 L of fixative continued for 20 minutes. While the perfusion was running, the left eye of each animal was injected in the limbal area with additional 0.5 mL of fixative. After that, the eyes were removed, cut along the equator, and placed in the same fixative for 10 days. A third animal R10103 (8-year-old, male) was included later. All procedures before starting the perfusion were similar to that of the first two animals, but the left eye of this monkey was treated as the eyes of animal R10019 and the right eye was treated as the eyes of animal R10026. The processing of the eyes during and after the perfusion was the same.

### Transmission Electron Microscopy (TEM) and Electron Tomography

Fragments were taken from different retinal regions of all the eyes. The fovea, easily located temporal to the optic disk as a thinner area of the retina with a diameter of about 2 mm, was used as the reference for all sampling. In greater detail, small pieces were taken from the fovea itself (within 1 mm from its center), the para/perifoveal area (between 1.5 and 3.0 mm from the center of the fovea) and the peripheral retina (>5 mm from the center of the fovea). Fragments were processed for TEM as previously reported.^25^

Ultrathin sections, cut along planes perpendicular or parallel to the retinal surface, were mounted on copper grids, stained and observed with a Philips 201 TEM. In other cases, 100- to 120-nm-thick sections were cut along planes parallel to the retinal surface, mounted on formvar-coated grids, stained, and covered with 10-nm colloidal gold particles as fiducial markers. Sections were then observed with a TEM Philips CM200 FEG supplied with a T-VIPS Tem-Cam F224 HD 2kxkx driven by the EM MENU 4 and EM-TOOLS software (T-VIPS, Millburn, NJ) for electron tomograms. Once a structure of interest was located, two tilt series of digital images were acquired at 27,500× with a range of ±60° and tilt steps of 1° along two orthogonal tilt axes. IMOD software for electron tomogram reconstruction and modelling was used to compute three-dimensional density maps starting from the images series.^26^ Tomograms, computed retrieving data from each perpendicular axis, were combined according to the standard IMOD protocol. Final tomograms, reconstructing a volume of 2048 × 2048 × 160 voxels (1228.8 × 1228.8 × 96 nm^3^), were viewed with the 3dmod 4.9 software (University of Colorado, Boulder, CO). Models of ICPSER were created by manually drawing membrane contours on consecutive 0.6-nm z-slices of the tomograms. Surface models were obtained using the same software.

### Morphometry and Statistical Analysis

Cells containing ICPSER were counted on ultrathin sections cut along planes parallel to RPE basement membrane so that the entire horizontal extension of the basal cytoplasm of each cell could be visualized. Cells were considered eligible for the count only when sectioned along their basal cytoplasm (Supplementary Fig. S1). One hundred RPE cells were checked for their ICPSER content on all specimens (fovea, para/perifovea, and peripheral retina) deriving from all the sampled eyes (3 closed eyes and 3 open eyes).

We explored the effect of eccentricity and light exposure on the number of ICPSER cells by fitting a Bayesian Poisson regression model to this dataset. We assumed that the number of ICPSER cells (per 100 cells) at the experimental condition *i, H_i_*, had a Poisson distribution with parameter *µ_i_*, which depended on the experimental conditions as follows:

(1) $$\log\;\mu_{i}=intercept+x_{i}^{T}\beta ,$$

where **x**_i_ = (*x_1_*.. *x_5_*, 1)^T^ comprises binary variables, or covariates, which represent all different combinations of eccentricity values with light exposure {dark fovea, dark perimacular, dark peripheral, light fovea, light perimacular, light peripheral}. The components of **x**_i_ take values *x_j_* = 1 if *j* = *i* or *x_j_* = 0 if *j* ≠ *i* for *j* = {1..5}; the value of *x_6_*, which is an arbitrarily chosen reference condition is fixed to 1 for all **x**_i_; **β** = (*β_1_*.. *β_5_*, 0)^T^ includes the model parameters which quantify the effect of experimental conditions.

To estimate the model parameters, we conducted Bayesian inference by Markov Chain Monte Carlo methods. The likelihood function for ICPSER cell counts at each condition and corresponding covariates is given *P*(*H_i_*|*x*_i_β) = Poisson(µ_*i*_), where *P*(^.^|^.^) denotes a conditional probability mass function. We used noninformative uniform prior distributions for the parameters *β_1_*.. *β_5_* and *intercept* in Equation 1.

Fitting diagnosis plots and posterior distributions for the parameters are provided in Supplementary Fig. S2 and Table S1. We used the posterior distributions to assess the significance of the effect of light exposure and retina region on ICPSER cell counts. Differences were considered significant when *P*{ *Pred_i_* > *Pred_j_ |* H } > 0.95, which denotes the posterior probability of the difference between predicted counts in experimental conditions *i* y *j* with H being the dataset of ICPSER cell counts used to fit the model.

Parameter inference and statistical analysis were performed with SAS 9.4 software (SAS Institute Inc., Cary, NC).

## Results

### General Arrangement of the SER in the RPE

**Figure 1.**
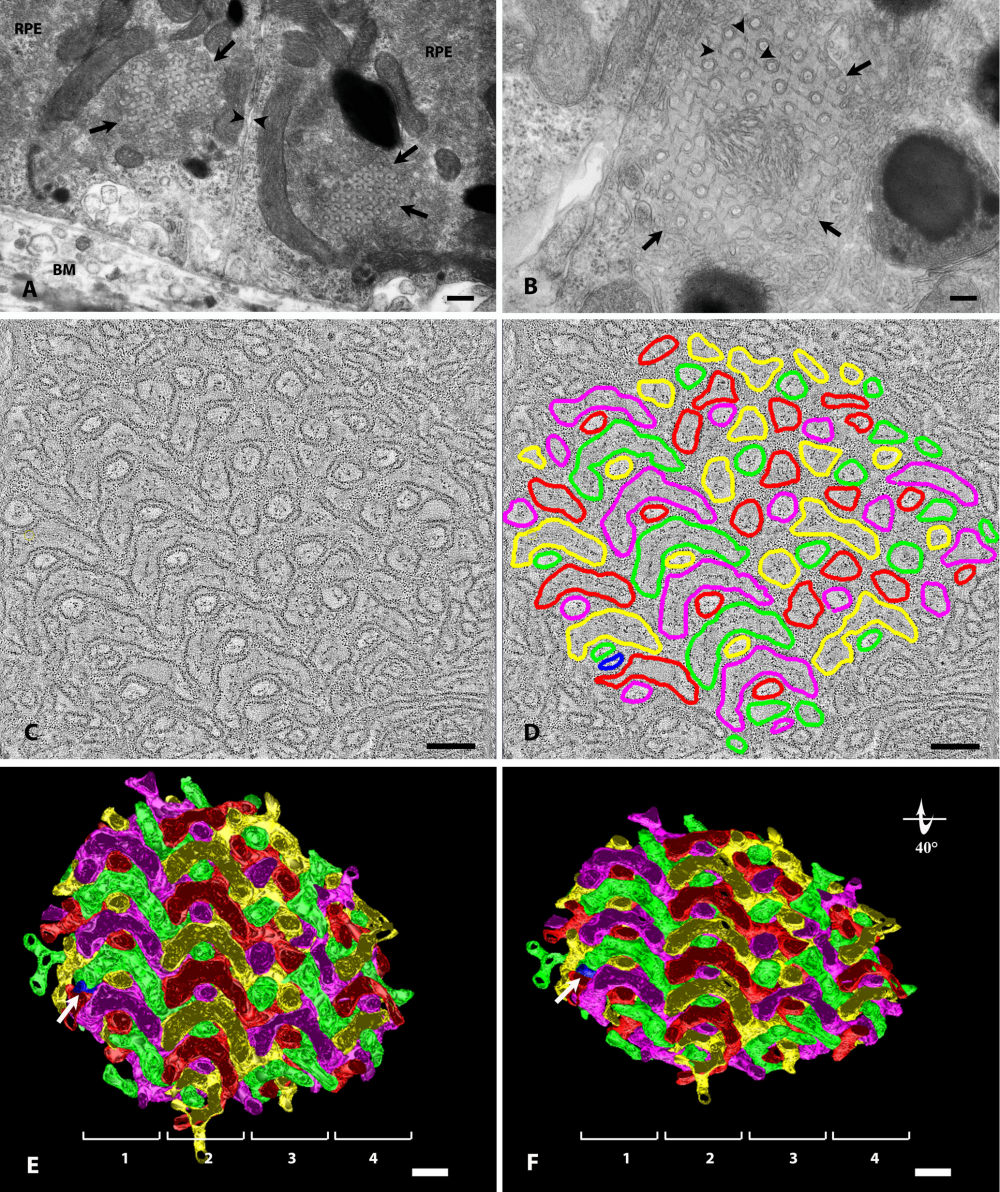
ICPSER in retinal pigmented epithelial cells of Rhesus Monkey. (**A**) Two adjacent retinal pigmented epithelial cells (RPE) are provided with ICPSER (*arrows*) at the corner of the lateral (*arrowheads*) and basal cell membranes. Monkey R10026. BM = Bruch's membrane. Magnification bar, 350 nm. (**B**) ICPSER (*arrows*) at higher magnification. The ICPSER shows a periodic array due to the checkerboard pattern of larger tubular structures surrounded by two concentric membranes and very small tubules (*arrowheads*) outlined by a single membrane. Larger and smaller tubules are apparently joined by straight filament-like elements. Monkey R10026. Magnification bar, 120 nm. (**C**) Electron tomography of an ICPSER unveils its complex nature. Thin axial slices (0.6 nm thick) show that membrane profiles follow a regular pattern realized by the alternation of larger C-shaped and smaller rounded tubules. This is particularly evident on the left where the section has a more favorable orientation. The distance between adjacent C-shaped tubules or between a C-shaped tubule and a rounded tubule is remarkably constant (8.5 nm). Monkey R10019. Magnification bar, 120 nm. (**D**) To generate a 3D-model of the ICPSER, membrane profiles have been retraced with different colors according to their continuity through the entire series of axial slices. Four major compartments, red, green, yellow and purple, can be identified. A fifth minor compartment (*blue*) can also be seen. Monkey R10019. Magnification bar, 120 nm. (**E**) A 3D rendering of the ICPSER. The model has been rotated on the *z*-axis to align compartments along the *x*- and *y*-axes. Four vertical rows (highlighted with numbered squared brackets) of different elements aligned along the *y*-axis can be appreciated as well as the alternation of C-shaped and rounded tubules (particularly as far as rows #1 and #2 are concerned). Periodicity in the model includes the regular sequence of the four major compartments that, however, follow inverted orders when comparing adjacent rows. A fifth minor compartment in blue (*arrow*) can also be seen as a single tubule traversing the ICPSER. Monkey R10019. Magnification bar, 120 nm. (**F**) A 3D rendering of the ICPSER. The model is the same shown in **E** though rotated by 40° on the *x*-axis. Magnification bar, 120 nm.

As previously reported,^17^^,^^18^ a large part of RPE cytoplasm was occupied by SER shaped as an irregular network of branched tubules. However, an ICPSER with a diameter of greater than 0.5 µm (between 0.5 and 1.5 µm) could be observed in 14.6% of RPE cells (263 ICPSER-containing cells/1800 cells). Interestingly, ICPSERs had a remarkably fix position within cells, being always located in the cytoplasmic corner encompassed by the basal and lateral domains of the plasma membrane (Figs. 1A–B).

### ICPSER in RPE Cells Shows a Periodic Structure

Before assessing whether they were somehow light-related structures, we explored the ICPSER structure on electron tomography. Once located an area containing an ICPSER, we acquired tomograms. Membrane profiles in a 0.6 nm-thick axial slice (Fig. 1C) outlined tubules with a diameter of 40 to 80 nm (60 nm on average). When tubules were very close to one another their opposing membranes run parallel at the constant distance of 8.5 nm.

Tracing membrane contours throughout the whole set of z-slices of the tomogram showed that ICPSER was made up by four major independent compartments of tubules that we colored in purple, green, red, and yellow (Fig. 1D). Three-dimensional modeling through the entire tomogram demonstrated that the compartments were arranged as an intricate weaving of tubules which, however, seemed to follow a highly ordered pattern (Figs. 1E–F). In particular, tubules in Figures 1E–F were arranged in four rows, each one containing elements belonging to each of the four major compartments. Rows 1 and 2 in Figure 1E were formed by a regular alternation of C-shaped tubules, apparently cut along their longitudinal axis, and by transversely cut tubules. Row 1 was made by the regular and periodic sequence from above downward of purple, red, green, and yellow compartments. Although with an inverted order (yellow, green, red, and purple from above downward), row 2 was similarly arranged. Rows 3 and 4 were apparently less organized because the slightly oblique cutting plane intercepted different regions of the ICPSER. However, even rows 3 and 4 showed the same arrangement on the reverse side of the model (Supplementary Movie S1)

**Figure 2.**
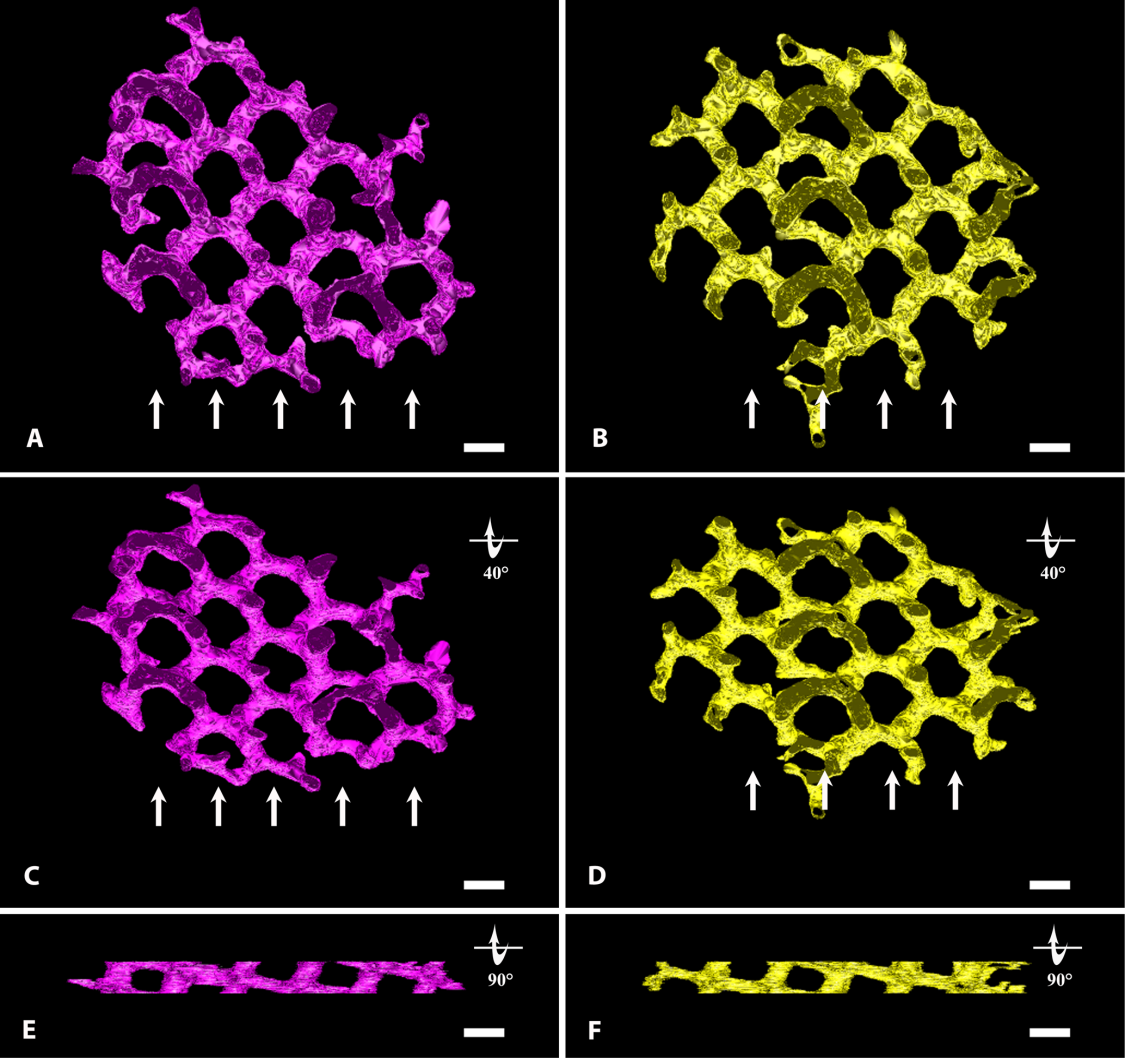
A 3D-rendering of ICPSER single compartments. (**A–B**) Two compartments (*purple* and *yellow*) have been isolated from the intricate weaving of tubules of the ICPSER shown in Figures 1E–F. The common plan for their construction is evident as they are made by the anastomoses of trijunctional tubules outlining parallel and staggered rows (*arrows*) of polygonal profiles. Magnification bar, 120 nm. (**C**–**D**) The same compartments shown in **A** and **B** and rotated by 40° on the *x*-axis allow to better appreciate the regular polygonal arrays outlined by the tubules. Magnification bar, 120 nm. (**E**–**F**) Rotation of the compartments shown in **A** and **B** by 90° on the *x*-axis changes the shape of the profiles outlined by the tubules that become quadrangular. Magnification bar, 120 nm.

To decipher in greater detail the ICPSER structure, we isolated and observed single compartments from various perspectives. By their comparison, it was evident that the four compartments were similarly built, although differently placed within the ICPSER (Fig. 2). Each compartment, composed by a meshwork of trijunctional very short tubules, outlined irregularly polygonal profiles on an *x*–*y* plane projection that were arranged in parallel but staggered rows. Polygonal profiles showed a center to center periodicity of 250 nm (Figs. 2A–B). In contrast, the rotation of the model by 90° on the *x*-axis changed dramatically the aspect of the specimen that showed a sequence of quadrangular profiles measuring approximately 60 × 100 nm (Figs. 2E–F, Supplementary Movie S2).

**Figure 3.**
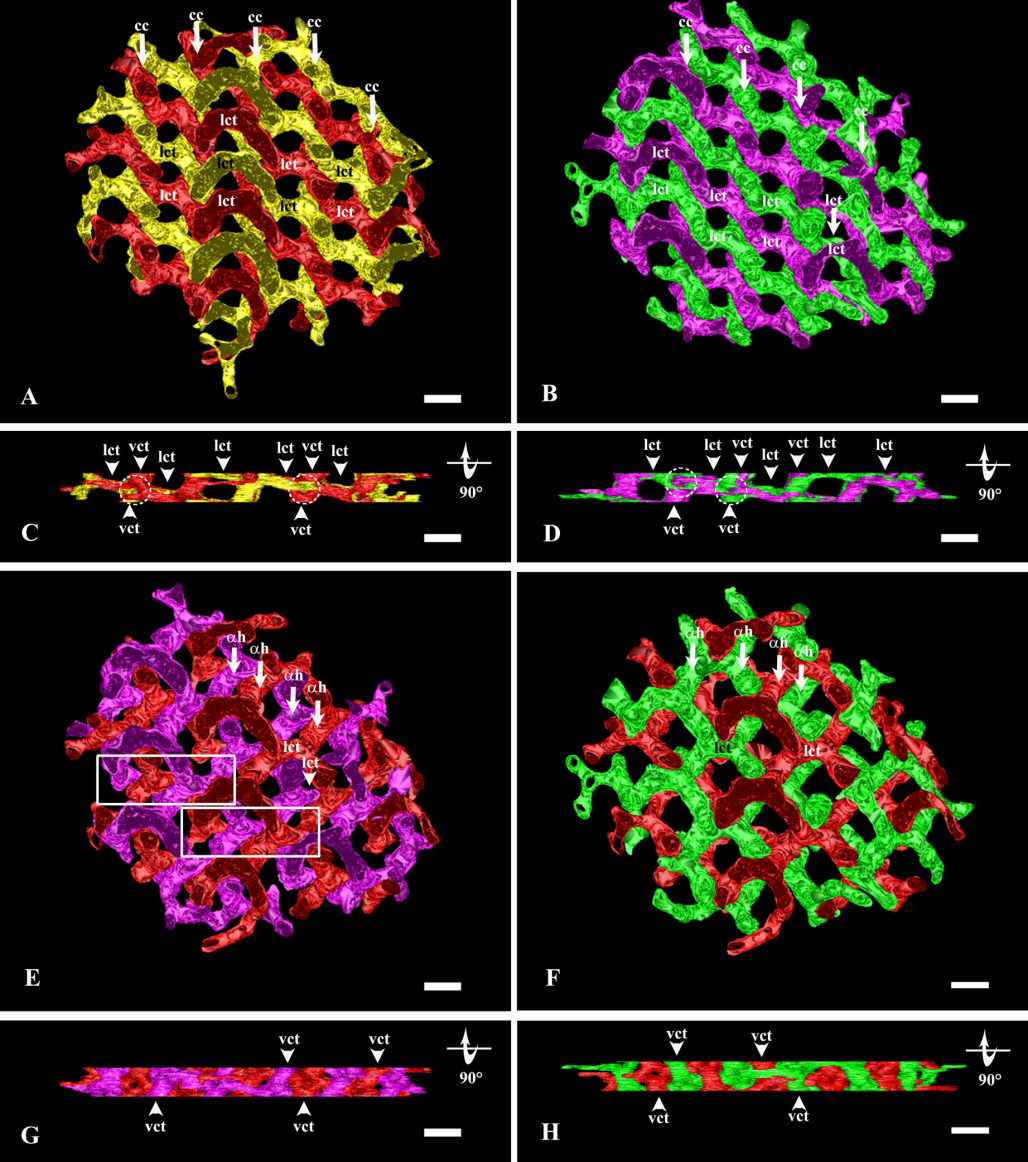
A 3D-rendering of couples of compartments isolated from the model shown in Figure 1. (**A**) ICPSER isolated yellow and red compartments. Tubules of both compartments develop α helices that wrap around each other to form coiled coils (cc) oriented along the *y*-axis. Coiled coils are joined by lateral connecting tubules (lct), which are placed along the *x*-axis. A regular alternation of red and yellow lateral connecting tubules can be observed along both the *y*-axis and the *x*-axis. Magnification bar, 120 nm. (**B**) Green and purple compartments are arranged similarly to the compartments shown in **A**. They too develop α helices that form coiled coils (cc) joined by lateral connecting tubules (lct). Coiled coils and lateral connecting tubules orientation is also alike the yellow and red compartments. Magnification bar, 120 nm. (**C**) The model shown in **A** has been rotated by 90°. The familiar profile already observed in Figures 2E–F can be observed even here as the yellow and red compartments are perfectly aligned. By this perspective, coiled coils are seen from one of their extremities (some of them have been highlighted with white circles). Vertical connecting tubules (vct) can be observed arising from coiled coils to join other coiled coils located at deeper and more superficial levels. Lateral connecting tubules (lct) can also be seen. Magnification bar, 120 nm. (**D**) The model shown in **B** has been rotated by 90°. The same profiles observed in **C** can be also seen here as the purple and green compartments are similarly aligned. The features described in **C** for the yellow/red compartments can be also observed here for the green/purple compartments. White circles = coiled coils; vct = vertical connecting tubules; lct = lateral connecting tubules. Magnification bar, 120 nm. (**E**) ICPSER isolated red and purple compartments. The couple of compartments, still arranged in parallel, are staggered. Tubules of both compartments develop α helices (αh), which are parallel and oriented along the *y*-axis. Lateral connecting tubules (lct) join the α helices of the same compartment. However, lateral connecting tubules and segments of the *y*-axis–oriented α helices also generate stretches of *x*-axis–oriented α helices which intertwine to form short coiled coils (framed areas). The couple of yellow and green compartments behaves in the same way (not shown). Magnification bar, 120 nm. (**F**) ICPSER isolated red and green compartments. They are arranged in parallel and aligned along the *x*-axis. Lateral connecting tubules and α helices (αh) can be also seen, although parallel α helices of these compartments do not wrap around each other and do not form coiled coils. The couple of yellow and purple compartments behaves in the same way (not shown). Magnification bar, 120 nm. (**G**) The model shown in **E** has been rotated by 90°. As the two compartments are not aligned along the *y*-axis, they fill the entire profile of the ICPSER. Nevertheless, vertical connecting tubules (vct) of both compartments can still be appreciated as well as the extremities of α helices (white circles) that can be identified at the cross-road of vertical and lateral connecting tubules. Magnification bar, 120 nm. (**H**) The model shown in **F** has been rotated by 90°. The same notes that have been made for the red/purple compartments applies for the red/green ones as well. Magnification bar, 120 nm.

The four compartments followed a parallel course. They were arranged as couples (red–yellow and purple–green) that were parallel and aligned along the *y*-axis (Figs. 3A–B), couples (red–purple and yellow–green) also parallel but staggered (Fig. 3E) and couples (red/green and yellow/purple) parallel and aligned along the *x*-axis (Fig. 3F).

At the ICPSER periphery, tubular compartments continued with the regular anastomosing SER (Fig. 3). Still at its periphery, ICPSER showed the outline of a fifth minor compartment that could be seen (Figs. 1D–F) as single tubules (from 1 to 3 depending on the sample) traversing the model and coursing parallel to one of the major compartments (Supplementary Fig. S3).

### ICPSER Is Formed by Coiled Coils of Tubular α Helices Joined Together by Connecting Tubules

**Figure 4.**
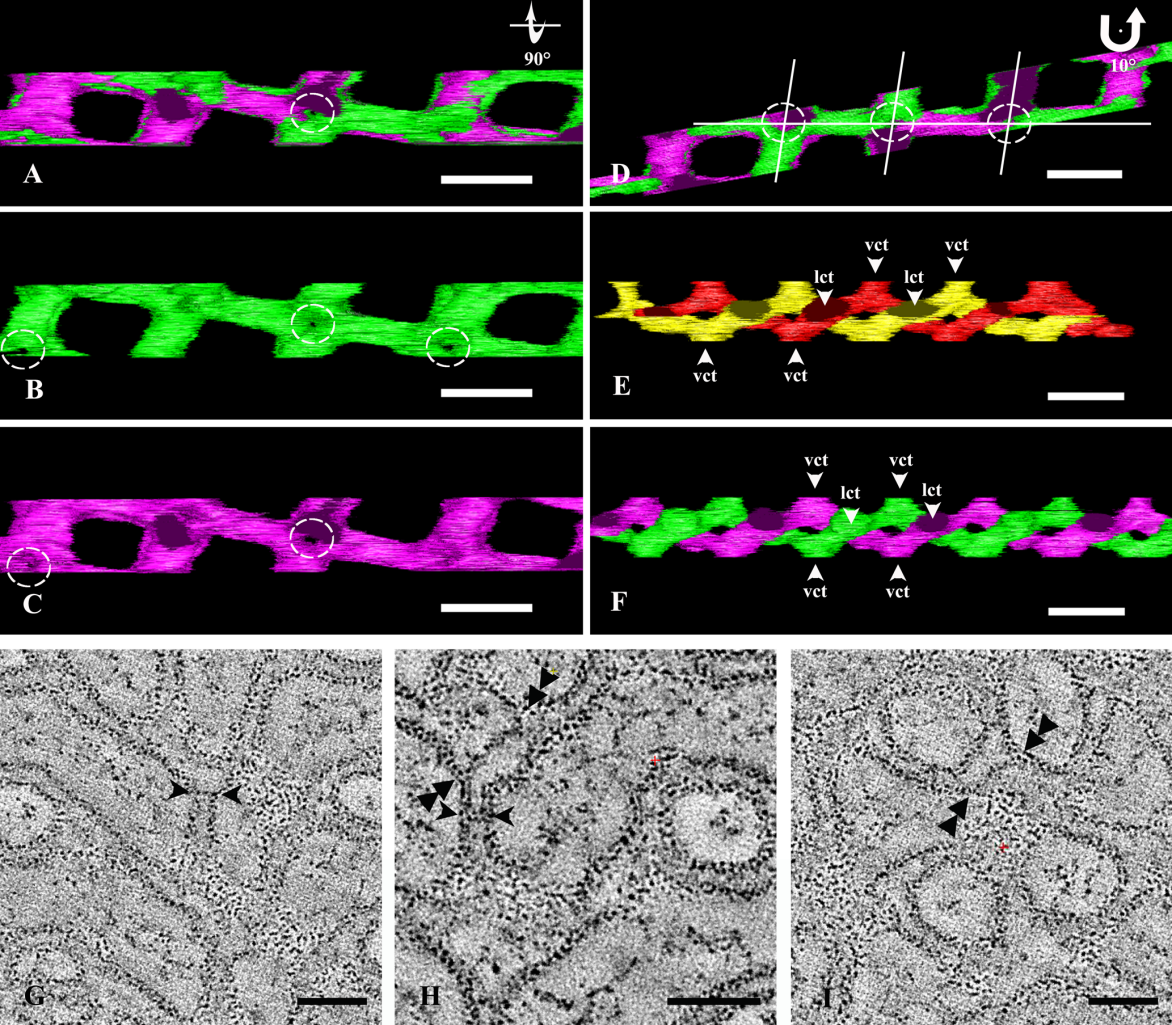
A 3D-rendering of couples of compartments (**A**–**F**). (**A**) The model shown in Figure 3B was rotated by 90° on the *x*-axis, partially clipped and enlarged. By this perspective, coiled coils are seen from one of their extremity. Magnification is high enough to glimpse a small eyelet at the center of one coiled coil (white circle) which corresponds to the axis around which the α helices wrap around each other. Magnification bar, 120 nm. (**B**–**C**) The compartments shown in **A** have been isolated. The eyelets (*white circles*) marking the central axis of the α helices are numerous. Vertical and lateral connecting tubules can be appreciated departing from the α helices. Magnification bar, 120 nm. (**D**) The model shown in **A** was tilted by 10° so that coiled coils, cut transversely (*white circles*), are now aligned along x planes. In this way, vertical connecting tubules are not perpendicular to coiled coils and lateral connecting tubules but they form an 80° angle. Magnification bar, 120 nm. (**E**) A single coiled coil has been isolated clipping the model shown in Figure 3**A**. The two α helices of the yellow and red compartments can be seen twisting around each other. Vertical connecting tubules (vct) and lateral connecting tubules (lct) are also visible arising at regular distance from the α helices. Magnification bar, 120 nm. (**F**) A single coiled coil formed by the green and purple compartments has been isolated clipping the model shown in Figure 3**B**. The same notes made for **E** also apply here. Magnification bar 120 nm. Intertubular filamentous framework (**G**–**I**). Delicate filamentous strands tether tubular compartment one to the other. Several examples are shown in encompassed between the black arrowheads. In some cases strands (encompassed by *double black arrowheads*) seems to be longer because they tether more distant tubules (**H**–**I**). Monkey 10026. Magnification bars, 60 nm.

The couples of parallel and aligned compartments along the *y*-axis outlined α helices wrapped around each other to form coiled coils of tubules (Figs. 3A–B, Supplementary Movie S3). Within the ICPSER, coiled coils belonging to the same couple of compartments lied one beside the other with a periodicity of 180 to 190 nm. When coiled coils or single α helices were cut perpendicularly, a tiny eyelet sometime became visible in their center, marking the axis around which α helices wound (Figs. 3C–D, 3G–H, 4A–D). Apparently, coiled coils were also aligned one over the other with a periodicity of about 200 nm. However, if coiled coils were opportunely placed along the *x*–*y* planes by a partial rotation of the model they did not result perfectly aligned vertically because those belonging to different planes were slightly shifted laterally (Fig. 4D). Coiled coils were not independent one from the other; they were joined by lateral and vertical connecting tubules (Figs. 3, 4A–F). Lateral connecting tubules arose at regular intervals (about 120–130 nm center to center) from coiled coils originating rigorously in an alternate fashion from the two α helices (Figs. 3A–B). Coiled coils were also joined by vertical connecting tubules with a center to center periodicity of about 120 to 130 nm. However, similar to the lateral ones, vertical connecting tubules belonged alternately to one or the other compartment. Connecting tubules directed downward and upward were roughly aligned one over the other, but belonged to different compartments, as to say they arose from different α helices (Figs. 4E–F). Because of the slight lateral shifting of the coiled coils located on different *x*–*y* planes, vertical connecting tubules formed an 80° angle with the lateral connecting tubules (Fig. 4D).

The thin cytoplasmic lamina intervening between tubules was frequently traversed by delicate strands that tethered together tubules belonging to different compartments. They could be found either joining the opposing extremities of two longitudinally cut C-shaped tubules or bridging transversely cut tubules with adjacent C-shaped tubules (Figs. 4G–I).

### ICPSER Frequency as a Function of Retinal Eccentricity and Light Exposure

**Figure 5.**
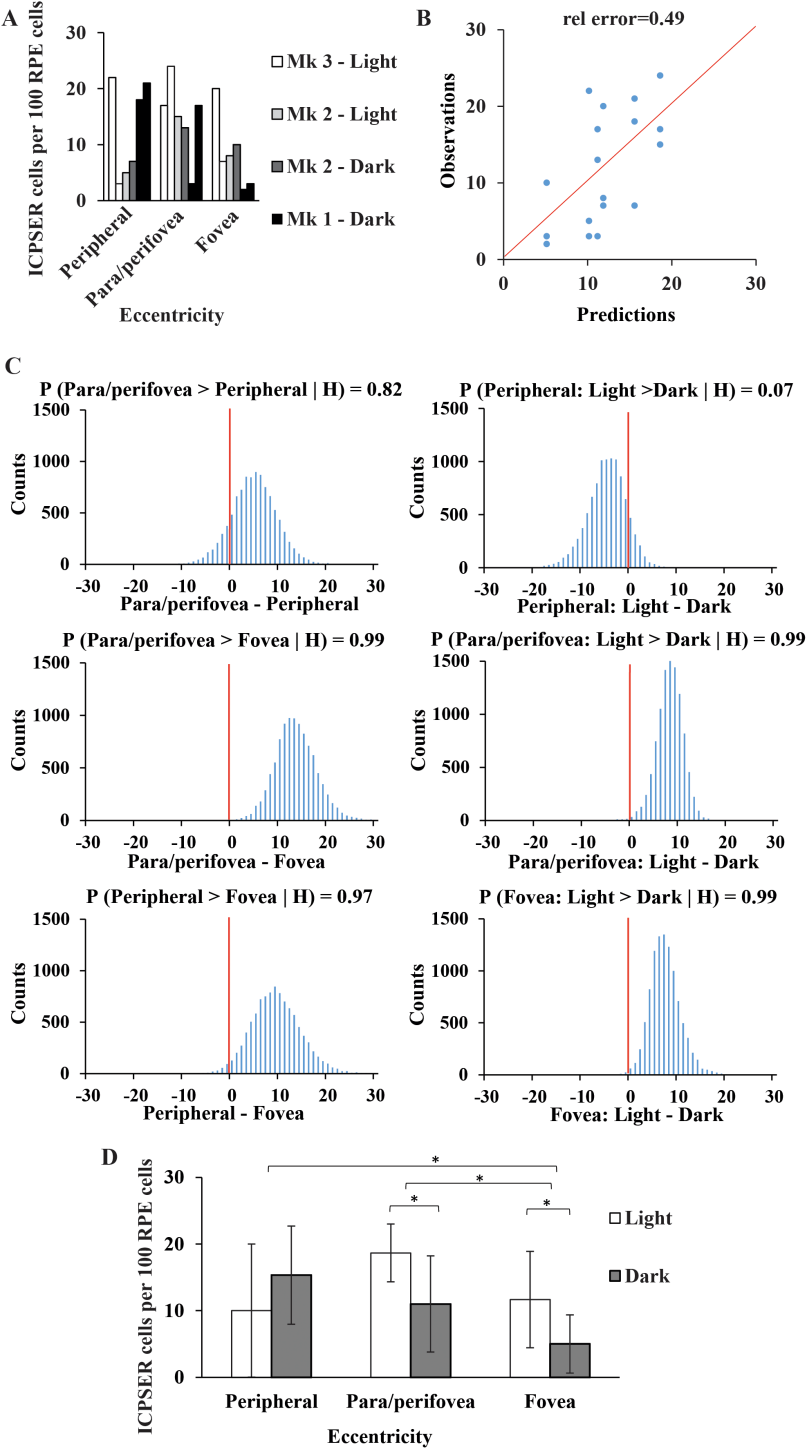
Modelling the effect of eccentricity and light exposure on ICPSER cell number. (**A**) ICPSER cell counts on retina regions of (3) monkeys’ eyes. (**B**) Scatterplot of observations in section A and predictions of a Bayesian Poisson regression model. The relative error was calculated as the average of the absolute relative differences. (**C**) The posterior distribution of the model predictions was used to assess the significance of the effect of light exposure and retina region on ICPSER cell counts. Differences were considered significant when *P*{ *Pred_i_* > *Pred_j_ |* H } > 0.95, which denotes the posterior probability of the difference between predicted counts in experimental conditions *i* y *j* with H being the dataset of ICPSER cell counts used to fit the model; **(D)** Bayesian inference indicated that ICPSER cells are significantly more abundant in the peripheral and para/perifoveal retina than in fovea and that their number in the fovea and para/perifoveal region increases significantly after light exposure.

To correlate ICPSER with RPE functions, we counted the number of cells containing the organelle in different areas of the retina (fovea, para/perifovea, and periphery) and in different conditions of light exposure (light vs darkness). We used a Bayesian Poisson regression model to investigate whether the observed cell counts in our experiments (Fig. 5A) could suggest that the region of the retina or the light exposure affected the number of cells containing ICPSER. Using Markov Chain Monte Carlo Methods, we could identify unambiguously the value of the model parameters and observed a good agreement between observed and fitted counts (Fig. 5B). Fitting diagnosis plots and posterior distributions for the model parameters are provided in Supplementary information (Supplementary Fig. S2 and Table S1). We next used the posterior distribution of lineal combinations of model predictions to investigate whether differences in cell counts between areas or upon light exposure were significant (Fig. 5C). Our Bayesian inference approach indicated that the experimental conditions affected the number of ICPSER-containing cells, which were significantly more numerous in the peripheral and para/perifoveal regions than in the fovea (Figs. 5C–D). We also quantified the effect of light exposure within each region and observed that in the fovea and in the para/perifoveal area the number of cells with ICPSER increased significantly in retinas exposed to light (Figs. 5C–D).

## Discussion

We have found that nonhuman primate *Macaca mulatta* RPE cells show a previously unknown domain of the SER characterized by an ICP. This domain apparently reminds of previously reported arrangements of organized SER (OSER) with cubic simmetry.^27^^–^^29^ Indeed, whereas OSER generates structures where membranes of adjacent tubules course parallel,^27^ membranes in ICPSER separate after a short tract; all compartments have a parallel course, but they are also interwoven. In addition, whereas OSER has few or any tripartite tubular junctions typical of anastomosing SER,^27^ ICPSER is just the result of the regular assembling of trijunctional tubules. More important, ICPSER differs from OSER in the higher complexity arranged in a hierarchy of three levels of organization. The first level consists of the regular meshwork of trijunctional tubules with a repeated branching pattern which defines each compartment. The second level is due to the coexistence of four parallel and independent meshworks of tubules (i.e., the four compartments). The third level of organization can be recognized by the relationships occurring among the four interwoven compartments whose α helices form tubular coiled coils. This complex arrangement of tightly packed tubules requires mechanisms of stabilization that likely take advantage of the intertubular tethers that we observed. Although the true nature of these tethers is unknown, similar filaments have been detected previously in areas of close relationships between organelles,^30^ and within the Golgi apparatus, where they also function as a compartment recognition system.^31^ Indeed, a system of compartment recognition is expected to be in place even in ICPSER to couple α helices of fellow compartments into coiled coils throughout the entire extension of the ICPSER.

ICPSER is present in about one RPE cell out of seven. Owing to its size and frequency, it is puzzling that ICPSER has never been spotted before. It is possible that ICPSER may be a species-specific feature and studies on RPE cells in vivo are certainly much less numerous in primates than in rodents. This consideration suggests that ICPSER may represent a special adaptation of RPE cells in primate foveate eyes and/or in diurnal animals.

Interestingly, ICPSER-containing cells increase in number in the area centralis upon light exposure, particularly in the para/perifovea where the difference with the number of ICPSER-bearing cells from dark-adapted retinas reaches statistical significance. Light exerts on RPE cells several effects, some of them detrimental when the level of exposure is too high. Light-induced dose-dependent alterations of mitochondria,^10^^,^^11^ formation of membranous whorls,^32^ disturbance of autophagy,^13^^–^^15^^,^^32^ and finally necrosis,^32^ all have been shown as consequences of intense illumination. High levels of light exposure even affect the RPE-dependent blood–retinal barrier. This outcome, which seems to be VEGF mediated, precedes and probably induces photoreceptor death.^24^^,^^33^ Photooxidation is certainly a key driver of light-induced RPE damage, mainly acting on pigments, which are major photosensitizers. For instance, in lipofuscin-loaded RPE cells, by a photooxidative-induced mechanism, blue light induces lysosomal enzyme leakage in the cytoplasm, activation of the inflammasome/caspase-1 cascade, and secretion of IL1β and IL18, which promote innate immune activation.^34^ Photooxidation of A2-E, a specific component of lipofuscin, decreases RPE autofluorescence, which, in turn, precedes RPE death when light exposure is intense enough.^35^^,^^36^

Because of the number of mitochondria, higher in cones than in rods, the high oxygen tension and the intense levels of focused light,^37^^,^^38^ primate cone-enriched fovea and its immediate surroundings (para/perifovea) are regions where the production of reactive oxygen species upon light exposure likely increases far more than in the peripheral retina. In the last years it has become increasingly evident that oxidative stress may also affect ER function and morphology. Oxidative stress can induce ER stress which, in turn, may activate proapoptotic signaling molecules.^39^ It is worthwhile to underline that short wave-length electromagnetic radiations, like UVA light, increases the expression of ER chaperones like calretinin, GRP78, and protein disulfide isomerase, which promotes apoptosis activating the PERK–eIF2α–ATF4–CHOP ER-mediated pathway.^40^ Ultimately, oxidative/ER stress may promote apoptotic loss of RPE cells as it occurs in AMD.^41^ Interestingly, the area centralis of the retina, where oxidative stress is likely more pronounced, is the very same area that shows a significant light-dependent increase in ICPSER-bearing cells suggesting that the appearance of ICPSER could be the manifestation of antioxidant responses or just a transient epiphenomenon of ongoing oxidative/ER stress. Surprisingly, the para/perifovea shows more ICPSER-containing cells compared with the fovea where light-induced oxidative stress should be more pronounced. This apparent paradox, however, can be the result of the concomitant presence of other antioxidant defenses. For instance, macular pigments like zeaxanthine and lutein, that locally decrease photo-oxidation by absorbing short wave-length blue light, are mostly concentrated in the fovea,^42^ just where ICPSER-bearing cells are less numerous. Even melanin, whose free radicals have protective effects scavenging reactive oxygen species in RPE,^43^ is more concentrated in the fovea.^44^

In other contexts, changes in ER morphology have been previously correlated with ongoing oxidative stress.^45^^,^^46^ The development of complex patterns of ER membrane foldings, as many cubic membranes and ICPSER essentially are, has been suggested previously as an antioxidant response that could shelter RNAs from oxidants.^45^ Although ICPSER is not a form of OSER, it shows some resemblance to cubic membrane arrangements as far as high membrane curvature is concerned and it might share some of the mechanisms required for its generation. Direct oxidation of lipids, for instance, induce the formation of cubic membranes,^47^ and might also contribute to ICPSER appearance.

In addition to exerting a protection to oxidative stress,^48^ RPE fulfils other functions including recycling of visual pigments.^49^ RPE SER is actually involved in recycling visual pigments as it harbors on its membrane several enzymes, including 11-*cis*-retinol dehydrogenase, lecithin:retinol acyltransferase and RPE65, which participate in the regeneration of all-*trans*-retinal into its 11-*cis*-retinal isomer.^50^^–^^52^ Pigment regeneration can be achieved by two different pathways. The first one, the canonical pathway, involves RPE cells and serves both cones and rods; the second one, the cone-specific pathway, engages Müller cells and, as the name points out, fulfils only cone demands.^49^^,^^53^ This finding suggests that the RPE workload for pigment recycling can be different depending on the number and type of photoreceptors that characterize specific retinal areas. In this respect, the fovea is a region highly enriched in cones, whereas the great majority of photoreceptors in the para/perifovea are rods.^54^ Such a diverse distribution of photoreceptors, and a likely greater involvement of RPE in visual pigment recycling in rod-enriched areas, could account for the higher number of ICPSER-bearing cells observed in the para/perifovea compared with the fovea.

In summary, this investigation has unveiled that *Macaca mulatta* RPE cells are characterized by a very complex SER domain, which is dynamically induced by light. The study has some intrinsic limitations; it was carried out on a limited number of animals belonging only to one species. It would, therefore, be presumptuous to make a generalization of these findings for other species. However, because in many ways human and Rhesus monkey's retinas are very similar, including the capacity to develop spontaneous AMD,^38^ comparable findings might be possible even for humans. Specific studies will be needed to address this issue, as well as to define ICPSER functions.

## Supplementary Material

Supplement 1

Supplement 2

Supplement 3

Supplement 4

Supplement 5

## References

[1] Thanos S, Heiduschka P, Romann I. Exposure to a solar eclipse causes neuronal death in the retina. *Graefe's Arch Clin Exp Ophthalmol*. 2001; 239: 794–800. 11760043 10.1007/s004170100362

[2] Kutty RK, Kutty G, Wiggert B, Chader GJ, Darrow RM, Organisciaki DT. Induction of heme oxygenase 1 in the retina by intense visible light: suppression by the antioxidant dimethylthiourea. *Proc Natl Acad Sci USA*. 1995; 92: 1177–1181. 7862656 10.1073/pnas.92.4.1177 PMC42661

[3] Algvere PV, Marshall J, Seregard S. Age-related maculopathy and the impact of blue light hazard. *Acta Ophthalmol Scand*. 2006; 84: 4–15. 16445433 10.1111/j.1600-0420.2005.00627.x

[4] Paskowitz DM, La Vail MM, Duncan JL. Light and inherited retinal degeneration. *Br J Ophthalmol*. 2006; 90: 1060–1066. 16707518 10.1136/bjo.2006.097436 PMC1857196

[5] Punzo C, Xiong W, Cepko CL. Loss of daylight vision in retinal degeneration: are oxidative stress and metabolic dysregulation to blame? *J Biol Chem*. 2012; 287: 1642–1648. 22074929 10.1074/jbc.R111.304428 PMC3265845

[6] Arnault E, Barrau C, Nanteau C, et al. . Phototoxic Action spectrum on a retinal pigment epithelium model of aged-related macular degeneration exposed to sunlight normalized conditions. *PLoS One*. 2013; 8: e71398. 24058402 10.1371/journal.pone.0071398 PMC3751948

[7] Marie M, Bigot K, Angebault C, et al. . Light action spectrum on oxidative stress and mitochondrial damage in A2E-loaded retinal pigment epithelium cells. *Cell Death Dis*. 2018; 9: 287. 29459695 10.1038/s41419-018-0331-5 PMC5833722

[8] Blasiak J, Pawlowska E, Szczepanska J, Kaarniranta K. Interplay between autophagy and the ubiquitin-proteasome system and its role in the pathogenesis of age-related macular degeneration. *Int J Mol Sci*. 2019; 20: 210. 10.3390/ijms20010210 PMC6337628 30626110

[9] Lakkaraju A, Umapathy A, Tan LX, et al. The cell biology of the retinal pigment epithelium. *Prog Ret Eye Res*. 2020; 78: 100846. 10.1016/j.preteyeres.2020.100846 PMC8941496 32105772

[10] Lauber JK. Retinal pigment epithelium: ring mitochondria and lesions induced by continuous light. *Curr Eye Res*. 1982; 2: 855–862. 7187642 10.3109/02713688209020022

[11] Del Olmo-Aguado S, Manso AG, Osborne NN. Light might directly affect retinal ganglion cell mitochondria to potentially influence function. *Photochem Photobiol*, 2012; 88: 1346–1355. 22364266 10.1111/j.1751-1097.2012.01120.x

[12] Schrader M, Wodopia R, Fahimi HD. Induction of tubular peroxisomes by UV irradiation and reactive oxygen species in HepG2 cells. *J Histochem Cytochem*. 1999; 47: 1141–1148. 10449535 10.1177/002215549904700906

[13] Remé CE, Wolfrum U, Imsand C, Hafezi F, Williams TP. Photoreceptor autophagy: effects of light history on number and opsin content of degradative vacuoles. *Invest Ophthalmol Vis Sci*. 1999; 40: 2398–2404. 10476808

[14] Chen Y, Sawada O, Kohno H, et al. Autophagy protects the retina from light-induced degeneration. *J Biol Chem*. 2013; 288: 7506–7518. 23341467 10.1074/jbc.M112.439935 PMC3597791

[15] Cheng KC, Hsu Y-T, Liu W, et al. The role of oxidative stress and autophagy in blue-light-induced damage to the retinal pigment epithelium in zebrafish in vitro and in vivo. *Int J Mol Sci*. 2021; 22: 1338. 33572787 10.3390/ijms22031338 PMC7866289

[16] Lynes EM, Simmen T. Urban planning of the endoplasmic reticulum (ER): how diverse mechanisms segregate the many functions of the ER. *Biochim Biophys Acta*. 2011; 1813: 1893–1895. 21756943 10.1016/j.bbamcr.2011.06.011 PMC7172674

[17] Porter KR, Yamada E. Studies on the endoplasmic reticulum. V. Its form and differentiation in pigment epithelial cells of the frog retina. *J Biophys Biochem Cytol*. 1960; 8: 181–205. 13737277 10.1083/jcb.8.1.181 PMC2224908

[18] Yorke MA, Dickson DH. Lamellar to tubular conformational changes in the endoplasmic reticulum of the retinal pigment epithelium of the newt, Notophthalmus viridescens. *Cell Tissue Res*. 1985; 241: 629–637. 4028144 10.1007/BF00214585

[19] Matthes MT, Basinger SF. Myeloid body associations in the frog pigment epithelium. *Invest Ophthalmol Vis Sci*. 1980; 19: 298–302. 6244230

[20] Tabor GA, Fisher SK. Myeloid bodies in mammalian retinal pigment epithelium. *Invest Ophthalmol Vis Sci*. 1983; 24: 388–391. 6682095

[21] Yorke MA, Dickson DH. Diurnal variations in myeloid bodies of the newt retinal pigment epithelium. *Cell Tissue Res*. 1984; 235: 177–186. 6538116 10.1007/BF00213738

[22] Tanito M, Yoshida Y, Kaidzu S, Ohira A, Niki E. Detection of lipid peroxidation in light-exposed mouse retina assessed by oxidative stress markers, total hydroxyoctadecadienoic acid and 8-iso-prostaglandin F_2α_. *Neurosci Lett*. 2006; 398: 63–68. 16442231 10.1016/j.neulet.2005.12.070

[23] Kurth I, Thompson DA, Rüther K, et al. Targeted disruption of the murine retinal dehydrogenase gene Rdh12 does not limit visual cycle function. *Mol Cell Biol*. 2007; 27: 1370–1379. 17130236 10.1128/MCB.01486-06 PMC1800705

[24] Cachafeiro M, Bemelmans A-P, Samardzija M, et al. Hyperactivation of retina by light in mice leads to photoreceptor cell death mediated by VEGF and retinal pigment epithelium permeability. *Cell Death Dis*. 2013; 4: e781. 23990021 10.1038/cddis.2013.303 PMC3763463

[25] Klug K, Herr S, Ngo IT, Sterling P, Schein S. Macaque retina contains an S-cone OFF midget pathway. *J Neusci*, 2003; 23: 9881–9887. 10.1523/JNEUROSCI.23-30-09881.2003 PMC6740873 14586017

[26] Kremer JR, Mastronarde DN, McIntosh JR. Computer visualization of three-dimensional image data using IMOD. *J Struct Biol*. 1996; 116: 71–76. 8742726 10.1006/jsbi.1996.0013

[27] Snapp EL, Hegde RS, Francolini M, et al. Formation of stacked ER cisternae by low affinity protein interactions. *J Cell Biol*. 2003; 163: 257–269. 14581454 10.1083/jcb.200306020 PMC2173526

[28] Almsherqi ZA, Kohlwein SD, Deng Y. Cubic membranes: a legend beyond the Flatland of cell membrane organization. *J Cell Biol.* 2006; 173: 839–844. 16785319 10.1083/jcb.200603055 PMC2063909

[29] Almsherqi ZA, Landh T, Kohlwein SD, Deng Y. Cubic membranes: the missing dimension of cell membrane organization. *Int Rev Cell Mol Biol*. 2009; 274: 275–342. 19349040 10.1016/S1937-6448(08)02006-6 PMC7105030

[30] Scorrano L, De Matteis MA, Emr S, et al. Coming together to define membrane contact sites. *Nature Comm*. 2019; 10: 1287. 10.1038/s41467-019-09253-3 PMC6427007 30894536

[31] Zhang X, Wang Y. GRASPs in Golgi structure and function. *Front Cell Dev Biol*. 2019; 3: 84. 10.3389/fcell.2015.00084 PMC4701983 26779480

[32] Pang J, Seko Y, Tokoro T. Processes of blue light-induced damage to retinal pigment epithelium cells lacking phagosomes. *Jpn J Ophthalmol*. 1999; 43: 103–108. 10340791 10.1016/s0021-5155(98)00073-2

[33] Jaadane I, Villalpando Rodriguez GE, Boulenquez P, et al. Effects of white light-emitting diode (LED) exposure on retinal pigment epithelium in vivo. *J Cell Mol Med*. 2017; 21: 3453–3466. 28661040 10.1111/jcmm.13255 PMC5706508

[34] Brandstetter C, Mohr LKM, Latz E, Holz FG, Krohne TU. Light induces NLRP3 inflammasome activation in retinal pigment epithelial cells via lipofuscin-mediated photooxidative damage. *J Mol Med*. 2015; 93: 905–916. 25783493 10.1007/s00109-015-1275-1 PMC4510924

[35] Sparrow JR, Boulton M. RPE lipofuscin and its role in retinal pathobiology. *Exp Eye Res*. 2005; 80: 595–606. 15862166 10.1016/j.exer.2005.01.007

[36] Morgan JIW, Hunter JJ, Masella B, et al. Light-induced retinal changes observed with high-resolution autofluorescence imaging of the retinal pigment epithelium. *Invest Ophthalmol Vis Sci*. 2008; 49: 3715–3729. 18408191 10.1167/iovs.07-1430 PMC2790526

[37] Hoang QV, Linsenmeyer RA, Chung CK, Curcio CA. Photoreceptor inner segment in monkey and human retina: mitochondrial density, optics, and regional variation. *Vis Neurosci*. 2002; 2: 395–407. 10.1017/s0952523802194028 12511073

[38] Pennesi ME, Neuringer M, Courtney RJ. Animal model of age related macular degeneration. *Mol Aspects Med*. 2012; 33: 487–509. 22705444 10.1016/j.mam.2012.06.003 PMC3770531

[39] Cao SS, Kaufman RJ. Endoplasmic reticulum stress and oxidative stress in cell fate decision and human disease. *Antioxid Redox Signal*. 2014; 21: 396–413. 24702237 10.1089/ars.2014.5851 PMC4076992

[40] Chen J-L, Hung C-T, Keller JJ, Lin H-C, Wu Y-J. Proteomic analysis of retinal pigment epithelium cells after exposure to UVA radiation. *BMC Ophthalmol*. 2019; 19: 168. 31375076 10.1186/s12886-019-1151-9 PMC6679551

[41] Dunaief JL, Dentchev T, Ying G.-S. The role of apoptosis in age-related macular degeneration. *JAMA Ophthalmol*. 2002; 120: 1435–1442. 10.1001/archopht.120.11.1435 12427055

[42] Davies NP, Morland AB. Macular pigment: their characteristics and putative role. *Prog Retin Eye Res*. 2004; 23: 533–559. 15302350 10.1016/j.preteyeres.2004.05.004

[43] Seagle B-LL, Gasyna EM, Mieler WF, Norris JR. Photoprotection of human retinal pigment epithelium cells against blue light-induced apoptosis by melanin free radicals from Sepia officinalis. *Proc Natl Acad Sci USA*. 2006; 103: 16644–16648. 17075067 10.1073/pnas.0605986103 PMC1636508

[44] Weiter JJ, Delori FC, Wing GL, Fitch KA. Retinal pigment epithelial lipofuscin and melanin and choroidal melanin in human eyes. *Invest Ophthalmol Vis Sci*. 1986; 27: 145–152. 3943941

[45] Deng Y, Almsherqi ZA. Evolution of cubic membranes as antioxidant defense system. *Interface Focus*. 2015; 5: 20150012. 26464785 10.1098/rsfs.2015.0012 PMC4590420

[46] Kong D, Liu R, Loiu J, et al. Cubic membranes formation in synchronized human hepatocellular carcinoma cells reveals a possible role as a structural antioxidant defense system in cell cycle progression. *Frontiers Cell Dev Biol*. 2020; 8: 617406. 10.3389/fcell.2020.617406 PMC7769198 33381509

[47] Sankhagowit S, Lee EY, Wong GC, Malmstadt N. Oxidation of membrane curvature-regulating phosphatidylethanolamine lipid results in formation of bilayer and cubic structures. *Langmuir*. 2016; 32: 2450–2457. 26866900 10.1021/acs.langmuir.5b04332 PMC6559366

[48] Plafker SM, O'Mealey GB, Szweda LI. Mechanisms for countering oxidative stress and damage in retinal pigment epithelium. *Inter Rev Cell Mol Biol*. 2012; 298: 135–177. 10.1016/B978-0-12-394309-5.00004-3 PMC3564215 22878106

[49] Bertelli E, Kondova I, Lagermans JAM. The retina. In: Bertelli E (ed). *Anatomy of the eye and human visual system*. Padova, Italy: Piccin-Nuova Libraria. 2019;187–247.

[50] Simon A, Romert A, Gustafson A-L, McCaffery JM, Eriksson U. Intracellular localization and membrane topology of 11-cis retinol dehydrogenase in the retinal pigment epithelium suggest a compartmentalized synthesis of 11-cis retinaldehyde. *J Cell Sci*. 1999; 112: 549–558. 9914166 10.1242/jcs.112.4.549

[51] Uppal S, Liu T, Poliakov E, Gentleman S, Redmond TM. The dual roles of RPE65 S-palmitoylation in membrane association and visual cycle. *Sci Rep*. 2019; 9: 5218. 30914787 10.1038/s41598-019-41501-w PMC6435699

[52] Mata NL, Tsin AT. Distribution of 11-cis LRAT, 11-cis RD and 11-cis REH in bovine retinal pigment epithelium membranes. *Biochim Biophys Acta*. 1998; 1394: 16–22. 9767084 10.1016/s0005-2760(98)00078-2

[53] Wang J-S, Kefalov V. The cone-specific visual cycle. *Prog Retin Eye Res*. 2011; 30: 115–128. 21111842 10.1016/j.preteyeres.2010.11.001 PMC3073571

[54] Provis JM, Penfold PI, Cornish EE, Sandercoe TM, Madigan MC. Anatomy and development of the macula: specialization and the vulnerability to macular degeneration. *Clin Exp Optom**.* 2005; 88: 269–281. 16255686 10.1111/j.1444-0938.2005.tb06711.x

