# Supplementary material for: Light-Induced Smooth Endoplasmic Reticulum Rearrangement in a Unique Interlaced Compartmental Pattern in Macaca mulatta RPE
Source: Invest Ophthalmol Vis Sci. 2021 Dec 30;62(15):32. doi: 10.1167/iovs.62.15.32 (PMC8727310; doi:10.1167/iovs.62.15.32)
Supplement: Supplement 1 [file iovs-62-15-32_s001.pdf]

Supplementary information

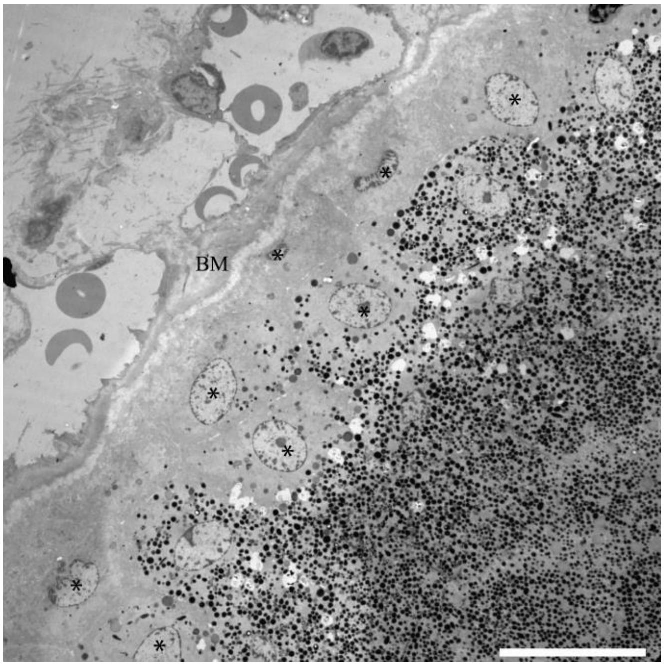

Supplementary Figure S1. **Cell eligibility for ICPSER count**. Ultrathin sections were cut along planes as much parallel as possible to the basement membrane. However, they could not be perfectly parallel because of the intrinsic curvature of the retina. Nevertheless, one or two rows of RPE cells closer to the basement membrane were sectioned along a plane passing through the lower half of the cytoplasm and therefore could be selected for the count. Cells eligible for the count in this field were marked with an asterisk. They always showed the nucleus (which is located in the basal cytoplasm) and were very poor of melanin granules (which gather to the apical part of the cells). All other cells were excluded because were cut along the apical cytoplasm (the nucleus could be no longer visible and they melanin granules were numerous). BM = Bruch's membrane. Magnification bar = 20 μm.

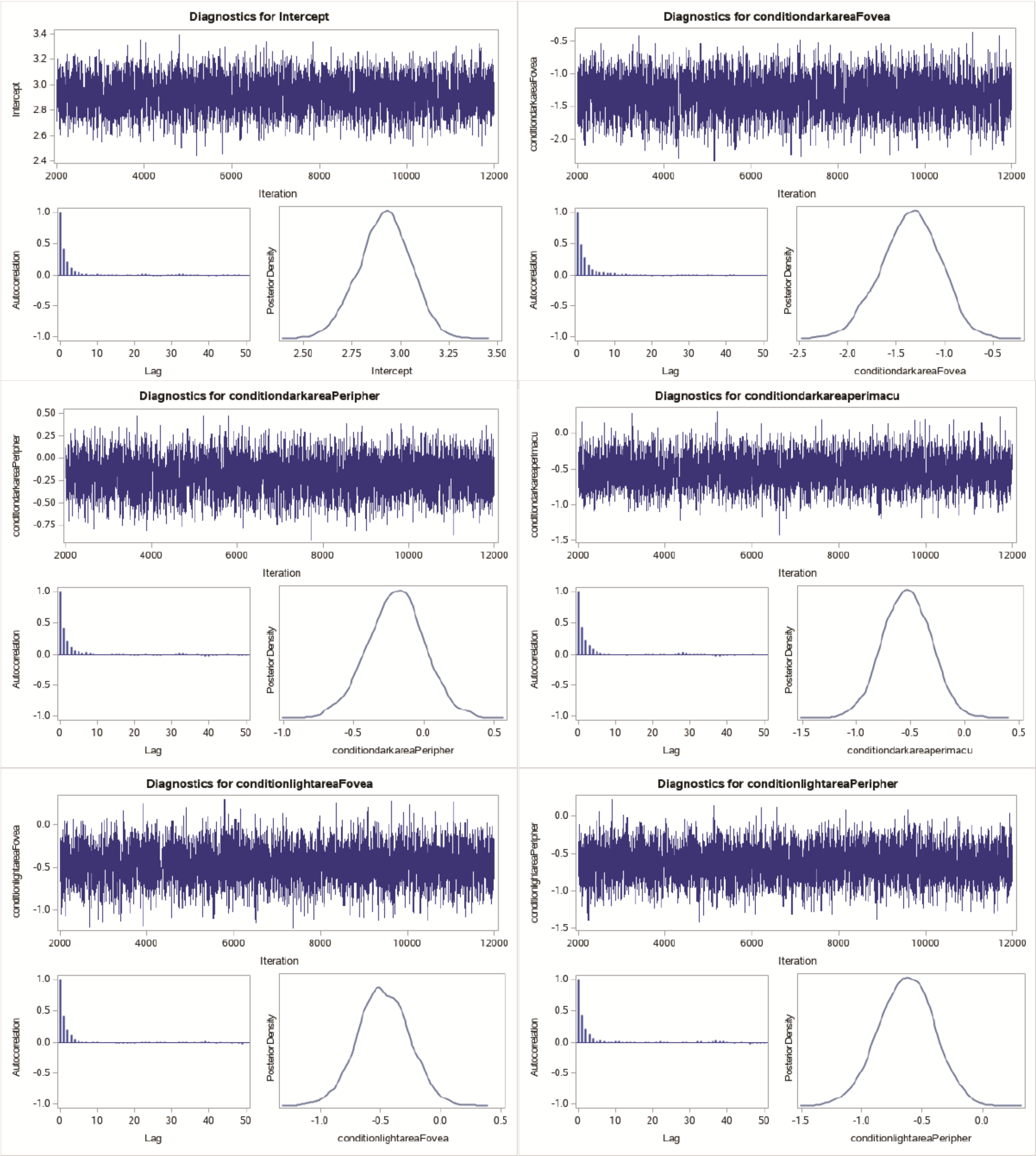

Supplementary Figure S2. Markov chain Mote Carlo (MCMC) diagnosis plots for the Bayesian estimates of model parameters. For each estimate, panels show a trace plot with values estimated in each iteration of the MCMC (top); autocorrelation functions (bottom left) and the posterior distribution (bottom right).

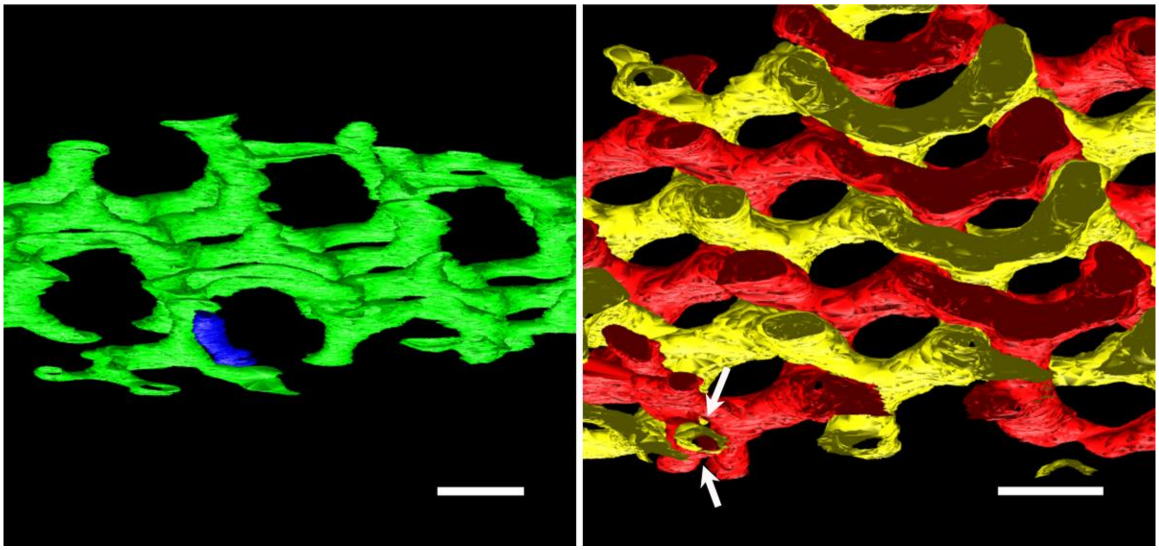

Supplementary Figure S3. **(A) Fifth compartment of the ICPSER.** The green and blue compartments of the HOSER have been isolated from the other components. The blue compartment appears rudimentary and traverses the HOSER as a small unbranched tubule parallel to one tubule of the green compartment. Magnification bar 120 nm. **(B) Peripheral continuity between major compartments**. 3D-modelling of the yellow and red compartments isolated from the other components of the OSER. At the periphery of the HOSER the two compartments are each other in continuity (arrows). The model has been clipped to uncover the continuity between yellow and red compartments.

Supplementary Movie S1. Rotation by 180° around the x axis of the HOSER model seen in Fig. 1E

Supplementary Movie S2. Rotation by 90° around the x axis of the model seen in Fig. 2B

Supplementary Movie S3. Rotation by 180° around the y axis of the seen in Fig. 3A
