# Supplementary material for: Light-Induced Smooth Endoplasmic Reticulum Rearrangement in a Unique Interlaced Compartmental Pattern in Macaca mulatta RPE
Source: Invest Ophthalmol Vis Sci. 2021 Dec 30;62(15):32. doi: 10.1167/iovs.62.15.32 (PMC8727310; doi:10.1167/iovs.62.15.32)
Supplement: Supplement 2 [file iovs-62-15-32_s002.pdf]

Table S1. Posterior mean, error and credible intervals inferred for model parameters by Markov Chain Monte Carlo methods.

Parameter	Experimental setting	Mean	Standard deviation	95% Highest posterior density interval	
Intercept		2.9158	0.1316	2.6684	3.1739
β_1	Dark* Fovea	-1.3327	0.2974	-1.9208	-0.7996
β_2	Dark* Peripheral	-0.1984	0.2	-0.5903	0.1915
β_3	Dark* Perimacular	-0.5333	0.2184	-0.9745	-0.1243
β_4	Light* Fovea	-0.4723	0.2114	-0.8968	-0.0716
β_5	Light* Peripheral	-0.6314	0.225	-1.0854	-0.2157
β_6	Light* Perimacular	0	-	-	-
